# Perovskite Random Lasers, Process and Prospects

**DOI:** 10.3390/mi13122040

**Published:** 2022-11-22

**Authors:** Lei Wang, Mingqing Yang, Shiyu Zhang, Chunhui Niu, Yong Lv

**Affiliations:** School of Instrument Science and Opto-Electronics Engineering, Beijing Information Science and Technology University, Beijing 100192, China

**Keywords:** random laser, perovskite, quantum dot, film

## Abstract

Random lasers (RLs) are a kind of coherent light source with optical feedback based on disorder-induced multiple scattering effects instead of a specific cavity. The unique feedback mechanism makes RLs different from conventional lasers. They have the advantages of small volume, flexible shape, omnidirectional emission, etc., and have broad application prospects in the fields of laser illumination, speckle-free imaging, display, and sensing. Colloidal metal-halide perovskite nanomaterials are a hot research field in light sources. They have been considered as desired gain media owing to their superior properties, such as high photoluminescence, tunable emission wavelengths, and easy fabrication processes. In this review, we summarize the research progress of RLs based on perovskite nanomaterials. We first present the evolution of the RLs based on the perovskite quantum dots (QDs) and perovskite films. The fabrication process of perovskite nano-/microstructures and lasers is discussed in detail. After that, the frontier applications of perovskite RLs are discussed. Finally, the challenges are discussed, and the prospects for further development are proposed.

## 1. Introduction

Random lasers (RLs), in which the optical feedback comes from the multiple scattering of the random media, have drawn much attention over the past decades [1,2,3,4,5]. In contrast to conventional lasers, RLs have many distinctive features, such as small volume, flexible shape, low fabrication cost, and omnidirectional emission. They show broad application prospects in the fields of high-brightness illumination, laser display, speckle-free imaging, and medical diagnostics [6,7,8,9,10,11]. The random lasing-like emission was first reported on from a solution containing rhodamine 640 and TiO_2_ particles by Lawandy in 1994 [12]. In 1999, the RL with extremely narrow peaks was observed from the ZnO powers by Cao et al. [13]. Since then, many light-emitting materials, such as organic fluorescent dye, oxide powders, semiconductor nanomaterials, and rare earth materials, have been used as the gain media of RLs [9,14,15,16,17,18,19,20,21,22,23,24,25]. 

In the past few years, colloidal metal-halide perovskites have become a global research hot spot [26,27,28,29,30]. Perovskites have a general chemical formula of ABX_3_, A_2_BX_5_, A_4_BX_6,_ and two-dimensional (2D) Ruddlesden–Popper of L_2_A_n−1_B_n_X_3n+1_, where A is CH_3_NH_3_ (MA), Cs, NH_2_-CH = NH_2_ (FA), etc.; B is Pb, Sn, Ge, etc.; X is Cl, Br, and I; and L is long organic chains [29,31]. They have attracted extensive attention in the fields of solar cells, light-emitting diodes, displays, and photodetectors because of the advantages of high carrier mobility, long carrier lifetime, high luminescence quantum yield (QY), narrow band emission, and simple solution fabrication processes [32,33,34,35,36,37,38,39]. 

The stability of halide perovskite materials is associated with the volatility of the A-site cation halides and the ligand anchoring groups. Typically, the thermal stability is in the order MAPbBr_3_ << FAPbX_3_ < CsPbBr_3_ [40]. Perovskite semiconductors, in the form of quantum dots (QDs), polycrystal thin films, and bulk single crystals, are also one kind of high-performance laser gain materials due to their high single/multiphoton absorption coefficient, low non-radiative recombination, high QY, and broad emission wavelength range [41,42,43,44,45,46,47,48,49,50,51,52,53,54,55,56]. In March 2014, Xing et al. first revealed that solution-processed perovskites were a new class of robust gain media with highly desirable characteristics [57]. They demonstrated the ultra-stable amplified spontaneous emission (ASE) from organic–inorganic halide perovskite MAPbI_3_ film with a low threshold of 12 µJ cm^−2^. The photostability of the MAPbI_3_ was assessed by monitoring the ASE intensity as a function of time under laser irradiation at room temperature. The ASE intensity was stable when the film was continuously irradiated for 26 hours. As a comparison, the typical ASE threshold of organic fluorescent dye-doped polymer films and CdSe/ZnCdS core/shell colloidal QD films were 30 µJ cm^−2^ and 90 µJ cm^−2^, respectively [14,58]. The low ASE threshold of MAPbI_3_ film was attributed to the low bulk defect density and slow Auger recombination of the film. Almost at the same time, the perovskite vertical-cavity lasing was achieved by embedding a layer of perovskite MAPbI_3−x_Cl_x_ between a dielectric mirror and a gold mirror [59]. Seven months later, the perovskite RL in planar 2D MAPbBr_3_ thin film was reported on by Giebink’s group [60]. The laser feedback comes from the scattering within the MAPbBr_3_ perovskite microcrystal network. In 2015, random lasing in the monodisperse cesium lead halide perovskite CsPbX_3_ QDs was first observed [61]. Since then, perovskite RLs have been intensively studied and achieved in various nanostructures such as QDs, polycrystal thin films, and single crystals. The representative studies of RLs from halide perovskites are summarized in Table 1. 

To date, the most studied perovskite RLs have been based on perovskite QDs and perovskite polycrystalline films. For perovskite QD RLs, multiple scattering media, such as nanoparticles, and wrinkled or nanoporous structures, are usually needed. The perovskite polycrystalline films can simultaneously serve as optical gain and multiple scattering media. The multiple scattering comes from the polycrystalline grain boundary and phase separation upon the phase transition. 

In this review, we overview the recent developments and achievements of RLs based on perovskite nanomaterials. We first present the evolution of the perovskite QD and perovskite film RLs in detail and then discuss their frontier applications. Finally, the challenges of perovskite RLs for application are discussed, and the prospects for further development are proposed. 

## 2. Perovskite QD RLs 

**Figure 1:**
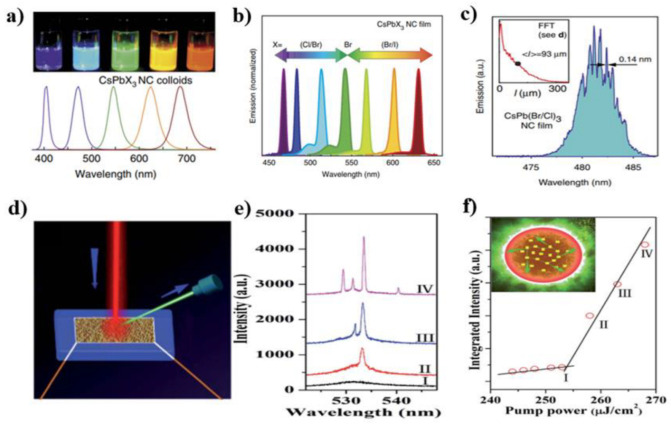
(**a**) Colloidal CsPbX_3_ NCs. (**b**) Spectral tunability of ASE via compositional modulation. (**c**) Single pump laser shot mode structure of random lasing from CsPb(Br/Cl)_3_ QD film. The inset shows path–length distribution averaged over 256 pump laser shots. The images (**a**–**c**) were adapted with permission from Ref. [61]. Copyright 2015 Springer Nature. (**d**) Schematic of CsPbBr_3_ NCs on a silicon substrate pumped via an 800 nm femtosecond laser. (**e**) Emission spectra of the CsPbBr_3_ NCs with different pump powers, showing the transition from spontaneous emission to lasing. (**f**) Integrated emission intensity as a function of pump density, showing the lasing threshold (254 mJ/cm^2^). Inset: Schematic diagram of the formation of a closed loop path for light through recurrent scattering in the samples. The images (**d**–**f**) were adapted with permission from Ref. [71]. Copyright 2020 The Royal Society of Chemistry.

In 2015, Kovalenko’s group reported on the ASE and lasing from the monodisperse all-inorganic perovskite CsPbX_3_ (X = Cl, Br, I) QDs for the first time [61]. The CsPbX_3_ QDs were synthesized by a one-step reaction between PbX_2_ and Cs-oleate in a nonpolar solvent. In this method, the PbX_2_ and ODE were first loaded into a flask and dried under a vacuum. Then the dried oleylamine and OA were injected under N_2_ protection. After the complete solubilization of PbX_2_ salt, the Cs-oleate solution was quickly injected. The reaction mixture was cooled 5 seconds later, obtaining the CsPbX_3_ QDs. Using these QDs as gain media, the authors observed the low-threshold ASE of 5–22 mJ/cm^2^ in the entire visible spectral range. The random lasing emission from CsPb(Br/Cl)_3_ QDs with narrow full width at half maximum (FWHM) of 0.14 nm was also obtained (Figure 1a–c). Later, Liu et al. studied the random lasing in self-assembled perovskite MAPbBr_3_ nanoparticles [62]. The MAPbBr_3_ QDs were synthesized by a one-step solution self-assembly method. Using a Ti:sapphire laser as a pump source, the single-photon and two-photon pumped RL with thresholds of 60 μJ/cm^2^, and 4.4 mJ/cm^2^ were realized. The near-field microscope image confirmed that the lasing actions come from the multiple scattering inside the perovskites. In 2016, Tang et al. prepared CsPbCl_3_, CsPbBr_3,_ and CsPbI_3_ QDs with tunable emission from 400 to 700 nm and studied their lasing properties [94]. The authors believed that the light scattering was caused by the disorder arrangement of CsPbX_3_ QDs in the films. 

The 2D materials with wrinkled structures have the advantages of stretchability and bendability. The wrinkled structure could trap the emitted light from the optical gain embedded in it. They are good scattering materials that can provide coherent optical feedback for the emitted light. In 2017, utilizing the reduced graphene oxide as wrinkled structures, Hu et al. designed a stretchable and wearable perovskite-based RL device [63]. In this device, the perovskite MAPbBr_3_ QDs, graphene film and polymer PDMS film were used as gain media, scatter and substrate, respectively. In the device fabrication process, a thin PDMS film was first stretched up to 100% of its original dimension. Next, the rGO films and MAPbBr_3_ QDs were spin-coated on the pre-strained PDMS substrate. Then the pre-strained PDMS was released to produce the wrinkled structure. Using a ps pulse laser as a pump source, the random lasing with threshold and FWHM of 10 µJ/cm^2^ and 0.4 nm were observed. This work provides a method to achieve flexible RL for wearable optoelectronic applications. Similarly, Roy et al. reported a perovskite RL by embedding MAPbBr_3_ QDs in the highly porous vertical-graphene nano-walls network [65]. The graphene networks could provide strong scattering and trap the photons effectively. The observed lasing showed an ultralow threshold of 10 nJ/cm^2^. Moreover, by coating Ag/SiO_2_ upon graphene networks, the threshold could be further lowered to 1 nJ/cm^2^. It is the lowest value reported so far. In 2019, Tang et al. distributed the CsPbBr_3_ QDs in solid honeycomb TiO_2_ nanotubes and successfully achieved room-temperature upconverted RL with a threshold of 9.54 mJ/cm^2^. The TiO_2_ nanotubes were used to enhance the optical multiple scattering effects [67]. 

In order to improve the stability of CsPbBr_3_ QDs, an accelerated transformation process from Cs_4_PbBr_6_ to defect-free CsPbBr_3_ QD was explored by Yang et al. [71]. In this work, the Cs_4_PbBr_6_ NCs were first synthesized by the hot injection method. Next, the deionized water was directly injected into a Cs_4_PbBr_6_ solution and irradiated under 365 nm light for 20 min. After being re-dispersed in toluene, the ultrahigh photo-and water-stable CsPbBr_3_ were obtained. It should be noted that the emission intensity could keep 92% after exposing them to 125 mL water for 45 days and could keep 93% when immersed in water and exposed to 365 nm illumination for 540 min. The observed lasing threshold was approximately 254 mJ/cm^2^, and the lasing could keep stable for more than 8.6 × 10^7^ excitation cycles (Figure 1d–f). 

**Figure 2:**
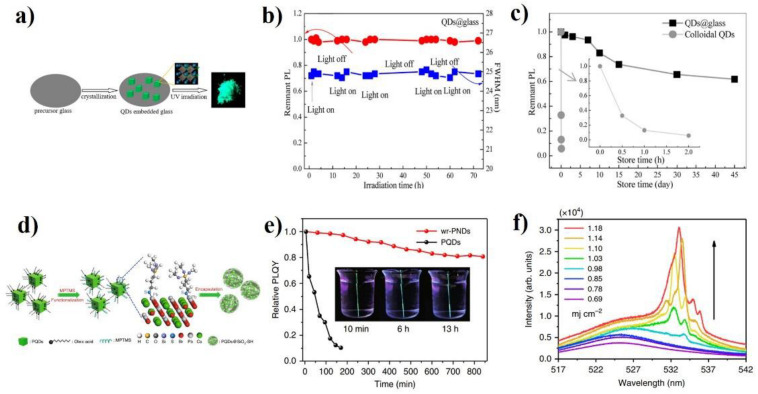
(**a**) Glass crystallization strategy to fabricate QD-embedded glass. (**b**) Photostability test of QDs@glass illuminated with 365 nm UV lamp: remnant PL intensity and the variation of FWHM as a function of the irradiation time. (**c**) Water resistance test by directly immersing QDs@glass in aqueous solution: remnant PL intensity vs. store time. The images (**a**–**c**) were adapted with permission from Ref. [64]. Copyright 2018 American Chemical Society. (**d**) Schematics of the synthetic procedure for making water-resistant perovskite QDs@SiO_2_ nanodots. (**e**) Relative PLQYs of the laser devices were based on water-resistant perovskite QDs@SiO_2_ nanodots (red dots) and pristine QDs (black dots), which were immersed in water for different periods. The insets are photographs of the nanodot-based laser in water taken at different times under UV light illumination. (**f**) Emission spectra of the water-resistant perovskite QDs@SiO_2_ nanodot-based RL device stored in water for 15 days. The images (**d**–**f**) were adapted with permission from Ref. [73]. Copyright 2020 Springer Nature.

The perovskite QDs possess high optical gain properties to produce room-temperature low-threshold lasers. However, they suffer from poor long-term stability due to their high sensitivity to UV irradiation, heat, water, and oxygen, which is a stumbling block to their practical applications [40]. The coating is regarded as an effective and facile way to solve this problem. In 2018, Yuan et al. developed an in situ nano-crystallization strategy to grow CsPbBr_3_ QDs directly into a more stabilized TeO_2_-based glass matrix for the first time [64]. In this method, the CsPbBr_3_ QDs@glass was designed with glass matrix compositions of TeO_2_-2Al_2_O_3_-14H_3_BO_3_-16ZnO-13Na_2_CO_3_ and perovskite components of Cs_2_CO_3_-PbBr_2_-KBr and CsBr-PbBr_2_. The precursor materials were first well mixed and ground into powders. After melting at 550–950 °C for 30 min and heating at 200–400 °C for 2–12 h, the QDs@glass was achieved. Those QDs showed superior photon, thermal, and water stabilities because of the effective protecting role of dense structural glass (Figure 2a–c). More importantly, those QDs distributed homogeneously inside the glass matrix, making them suitable to act as disorder gain media to realize RL. Under pumping by 800 nm fs laser, the RL emission with FWHM of 1nm and corresponding threshold of ∼200 μJ cm^−2^ was achieved at 77 K. This work presents a new route to fabricate perovskite QDs with excellent stability for lasing applications. Later, Liu et al. fabricated CsPbBr_3_ QDs embedded in microsized SiO_2_ (CsPbBr_3_-SiO_2_) spheres and realized a stable upconverted hybrid whispering-gallery-mode and random lasing in these spheres [66]. The RL threshold was approximately 65 μJ/cm^2^. The coherent feedback comes from the internal reflection between SiO_2_ and air. Li’s group proposed a Pb-S bonding approach to fabricate water-resistant CsPbBr_3_ QDs@silica nanodots [73]. These nanodots could keep emissions for six weeks when they were dispersed in water. Using these nanodots as gain media, the uncovering WG mode lasing and random lasing were also observed (Figure 2d–f). More importantly, the lasing emission could operate after the nanodots were dispersed in water for up to 15 days. In contrast to previous works, the RL showed great water stability. Jin et al. fabricated CsPbI_3_ QDs embedded into a glassy matrix through melt-quenching and glass crystallization methods [78]. The QD glass meets the requirements of oxygen, thermal, and water stability. After immersing the QD glass in water for one week, the PL intensity was maintained at 85%, and random lasing emission could still be achieved. 

Wang et al. further synthesized a dual-phase mixed CsPbBr_3_ QDs@glass@styrene-ethylene-butylene-styrene (SEBS) nanocomposite film [22]. To fabricate this film, the CsPbBr_3_ QDs@glass was first grounded entirely into micron-fine powders and mixed with the prepared SEBS-toluene solutions. After that, the mixed solution was tiled on a circular glass substrate, allowing the film to solidify. The nanocomposite film exhibited a high QY of ∼50%, outstanding UV/thermal/water resistance, and environmental friendliness. Upon pumping by an fs laser with a wavelength of 800 nm, the random upconverted laser was achieved. The RL from the surface ligand-modified CsPbBr_3_ QDs adhering on the surface of the micro SiO_2_ sphere was also reported [79]. 

Apart from the CsPbBr_3_ QDs, the FAPbBr_3_/SiO_2_ composites were also studied. In 2020, Yang et al. successfully synthesized the FAPbX_3_ QDs adhering to the surface of amino-mediated silica (A-SiO_2_) spheres using the ligand-assisted reprecipitation (LARP) method and studied the up-conversion random lasing [68]. The fabricated process is shown as follows. First, the FAX, PbBr_2_, A-SiO_2_ spheres, oleylamine, and oleic acid ligands were mixed in DMF, forming a precursor solution. Then, the precursor solution was injected into chloroform. Within seconds, bright green-emitting FAPbBr_3_/A-SiO_2_ composites were formed. The as-prepared FAPbBr_3_/A-SiO_2_ composites possess high photoluminescence with a QY of 80%. Using an 800nm fs laser as a pump source, the up-conversion RL with a low lasing threshold of 413.9 μJ/cm^2^ and high-quality factors of 1307 was achieved. Meng’s group developed a new approach to realize perovskite RLs by in situ precipitating perovskite QDs in metal−organic frameworks (MOFs) [75]. MOFs are a class of porous crystalline materials. The well-defined and interconnected nanoporous channels could effectively prevent the aggregation of gain media and serve as excellent scattering centers for RLs. In this work, Pb-MOFs, which acted as both the source of lead and the porous template, were utilized as a matrix. The high-density perovskite QDs were directly fabricated via the one-step process. The Pb^2+^ ions in Pb-MOFs provided sufficient binding sites, thus ensuring the generation of high-density gain in MOFs. Using this method, they fabricated MAPbBr_3_ QDs@Pb-MOFs, MAPbBr_x_Cl_3−x_@Pb-MOFs, and MAPbBr_y_I_3−y_@Pb-MOFs. The low-threshold multicolor RLs in the visible spectral range were achieved. Such RLs make full use of the merits of the MOFs of a high refractive index and a broad transmission window. Applying these RLs as an illumination source, speckle-free optical imaging was also demonstrated. Gao et al. proposed a flexible perovskite RL by depositing CsPbBr_3_ QDs onto Ni porous foam [77]. Ni porous foam has a 3D heat-conductive metallic structure of voids and provides strong light scattering between the voids. The laser emission could be tuned by controlling the surface morphologies of the Ni foam through deformation. 

The most studied perovskites, such as CsPbBr_3_ and FAPbBr_3_, are composed of three-dimensional interconnected octahedral-structural units. For CsPbBr_3_ perovskites, the [PbBr_6_]^4−^ octahedra units have three-dimensional interconnectivity, while Cs^+^ ions fill the voids between them. They typically suffer from severe PL quenching. In contrast to CsPbBr_3_, Cs_4_PbBr_6_ has the structure of the [PbBr_6_]^4−^ octahedra being entirely separated, making the excitons strongly localized [95]. It gives rise to large exciton binding energy, which is beneficial to preventing the PL quenching in solid forms of Cs_4_PbBr_6_. In 2020, Liu et al. demonstrated the capability of perovskite Cs_4_PbBr_6_ monolith ceramics to trigger low-threshold single-/two-photon-pumped RL for the first time [70]. The perovskite Cs_4_PbBr_6_ monolith ceramics were fabricated by a high-temperature vapor-phase drop-casting approach. Briefly, the materials were first mixed and put into an alumina crucible. It was heated to 1200 °C for 20 min and then quickly drop cast and cooled. After that, the centimeter scales of Cs_4_PbBr_6_ monolith ceramics were obtained. The thresholds of single-photon-pumped and two-photon-pumped RL were ∼25.98 μJ/cm^2^ and ∼719 μJ/cm^2^, respectively. The lasing emission verified the green PL observed in the developed Cs_4_PbBr_6_ ceramics coming from intrinsic defects instead of CsPbBr_3_ contaminations. Plasmonic nanolasers provide an opportunity for expanding lasers in subwavelength applications. In 2021, Xing et al. reported a localized surface plasmon resonance (LSPR) supported CsPbBr_3_ QD random lasing from the Ag nanowire embedded CsPbBr_3_ QD films [76]. The observed lasing arises from the interactions between the QDs and Ag nanowires. When the pump light focused on a single Ag nanowire, the plasmonic-waveguide laser with a Q factor of 1300 was also observed. 

Optical-pumping QD lasers are limited in practical application because of the high requirement for ultra-fast laser light sources. Electron beam (EB) pumping could partially solve this problem. Zhu et al. studied the CsPbBr_3_ RL using EB pumping [69]. In this work, the CsPbBr_3_ QD films were used as the gain media. The designed EB pumping laser device included an external trigger light source, Au cathode, microchannel plate, QD films, and TIO conductive glass. In this laser device, the initial photoelectrons from the Au photocathode were first triggered by a UV signal, and then the electron beam was amplified by a microchannel plate (MCP), obtaining the high-energy electron beam to pump the CsPbBr_3_ QD films. The voltage was applied to the microchannel plate and the ITO glass, forming an extra electric field (EF). When the voltage was strong enough, the low-energy particles could absorb sufficient energy to jump to high energy levels, resulting in massive photon emissions. At low voltage, the CsPbBr_3_ QD films showed spontaneous emission. When the external voltage exceeded 3 kV, a spike appeared in the emission spectrum. With further increased external voltage to 5 kV, sharp RL peaks appeared with an FWHM of 0.9 nm. The EB pumping method has the advantages of high efficiency and high stability. It provides a compact RL device for the integrated application. 

## 3. Perovskite Film RLs 

**Figure 3:**
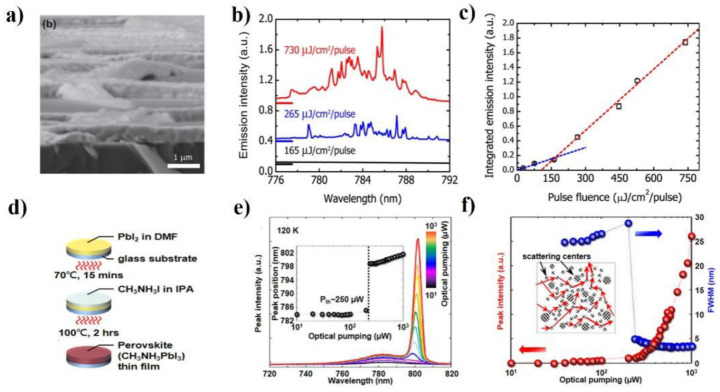
(**a**) Cross-sectional SEM image of the perovskite network. (**b**) Emission spectra are collected below, near, and above the lasing threshold. (**c**) Integrated emission intensity as a function of excitation pulse fluence. The lasing threshold is 195 ± 30 μJ/cm^2^/pulse. The images (**a**–**c**) were adapted with permission from Ref. [60]. Copyright 2014 AIP Publishing LLC. (**d**) Schematic diagram of the perovskite MAPbI_3_ films prepared in solution processes. (**e**) PL emission spectra of the fabricated perovskite films, photo-excited at different optical-pumping powers. (**f**) L-L curve plotted on a log-linear scale and the corresponding linewidths as a function of pumping powers. The images (**d**–**f**) were adapted with permission from Ref. [96]. Copyright 2014 AIP Publishing LLC.

The random lasing from perovskite films was first reported by Dhanker in 2014 [60]. In this work, the planar 2D MAPbBr_3_ film was used as the gain medium. In the fabrication process, the precursor was first prepared by mixing MAI and PbI_2_ in a DMF solution. The solution was then spin-coated onto glass and annealed at 115 °C on a hot plate. After annealing, the MAPbBr_3_ film formed. The laser device was fabricated by encapsulating the film using a glass coverslip and an edge bead of ultraviolet-curable adhesive. Using an ns laser as a pump source, the authors observed bright RL spots with thresholds of 200 μJ/cm^2^. The linewidth of the laser peaks was less than 0.5 nm (Figure 3a–c). They proved that the RL originated from a combination of waveguiding and scattering within the perovskite microcrystal network. Soon after, Lu’s group studied the temperature-dependent lasing characteristic of solution-processed MAPbI_3_ films [96]. To fabricate this film, the authors first spin-coated the as-prepared PbI_2_ solution on a glass substrate and dried it at 70 °C for 15 min, obtaining yellow PbI_2_ film. Next, the PbI_2_ film was covered with an MAI solution and annealed at 100 °C for 2 h to form the dark-brown MAPbI_3_ perovskite layers of around 300 nm in thickness. The lasing emission from these films could sustain to a near room temperature at 260 K (Figure 3d–f). The multiple scattering comes from the polycrystalline grain boundary and/or phase separation upon the phase transition. Using a similar fabrication method, this group further demonstrated a controllable MAPbI_3_ perovskite RL with a low threshold [80]. In 2018, Safdar et al. fabricated uniform MAPbI_3_ films on glass by a simple solution deposition step and demonstrated a high-performance room-temperature RL [83]. The laser threshold was approximately 10 μJ/cm^2^. 

**Figure 4:**
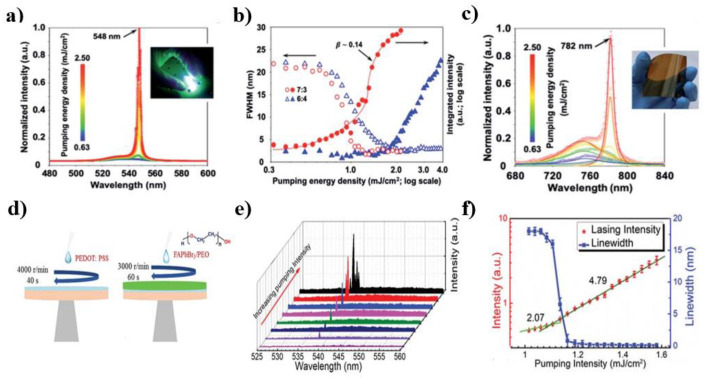
(**a**) Lasing characteristics of MAPbBr_3_ organic–inorganic metal-halide perovskites. (**b**) The light–light curves extracted from the films were synthesized at different volume ratios. (**c**) Lasing characteristics of solvent-engineered MAPbI_3_ perovskite thin films. The images (**a**–**c**) were adapted with permission from Ref. [88]. Copyright 2020 The Royal Society of Chemistry. (**d**) The preparation of the PEDOT:PSS and the FAPbBr_3_/PEO layers. (**e**) Two-photon pumped random lasing spectra versus pumping intensity. (**f**) Two photon-pumped random lasing intensity and FWHM versus pumping intensity. The images (**d**–**f**) were adapted with permission from Ref. [81]. Copyright 2018 The Royal Society of Chemistry.

The random lasing behavior of solution-processed perovskite films results from the random scattering in films. The substrate, morphology, and structural configuration of the films can affect their optical and especially the lasing performance. They can be controlled by manipulating the fabrication processing parameters, such as the ratios of pin-coating speed, engineered solvent mixtures, and substrate. Weng et al. compared the random lasing properties of MAPbBr_3_ perovskite films on different substrates [97]. The average thickness of the perovskite films was approximately 5 µm. The lasing threshold of the perovskite films on the patterned sapphire substrate was one order lower than on the fluorine-doped tin oxide substrate. The giant threshold reduction is attributed to the enhanced random scattering of light at the perovskite/sapphire interface. Hu et al. further prepared the MAPbBr_3_ multi-crystalline thin films on indium–tin oxide (ITO)-coated glass, fused silica (FS), and tricyclo decanedimethanol diacrylate (ADCP) substrates and studied the influence of substrates on the morphological and random lasing properties [93]. They found that the MAPbBr_3_ film on the FS substrate produced the thickest wrinkle-like stripes, whereas that on the ADCP substrate exhibited the thinnest modulation. The MAPbBr_3_ film on the ADCP substrate had larger crystal particles, implying stronger optical scattering. Hong et al. proposed an optimized anti-solvent dripping fabrication process. In this process, the GBL and DMSO were used as the solvent [88]. The thickness of the perovskite films was 100 nm. By tuning the volume ratios of solvent mixtures, the crystallization rate and structural morphology of grain boundaries could be controlled. Experiential results showed that the MAPbBr_3_ films exhibited the lowest lasing threshold and highest lasing intensity at the solvent volume ratio of 7:3 (Figure 4a–c). Moreover, the optimized lasing showed great long-time reliability with 12 × 10^5^ pulses without any sealing package. Mallick et al. introduced a polystyrene passivation layer on MAPbBr_3_ perovskite films and studied the influence of polystyrene mixing concentrations on random lasing properties [89]. The passivation layer could improve the stability and reduce the non-radiative recombination of the MAPbBr_3_ film. The perovskite–polystyrene (30 wt.%) based RL showed the lowest lasing threshold and best contrast to noise ratio values in speckle-free imaging. 

The multiphoton absorption process features merits of large penetration depth, little photodamage, and photobleaching. In 2018, Weng et al. synthesized a high-quality MAPbBr_3_ perovskite thin film through a one-step spin-coating method and demonstrated a three-photon (3P) pumped perovskite RL with fs laser pumping at 1300 nm [82]. The respective threshold was estimated to be 27 mJ/cm^2^. Despite the low conversion efficiency of the 3P absorption process, the 3P excitation was found to be effective in reducing the RL threshold. It was explained by the larger penetration depth and the more effective excitation of internal perovskite lattices for 3P absorption processes. Xu et al. studied the two-photon pumped random lasing from the FAPbX_3_ perovskite films [81]. In this work, FAPbBr_3_/polyethylene oxide (PEO) composited films with better moisture and temperature stability were fabricated by the solution processing method. The lasing threshold was approximately 1.1 mJ/cm^2^ when pumped by an ns pulse laser with an excitation wavelength of 1064 nm (Figure 4d–f). The lasing in the composite film was from the multiple scattering of light among the PEO bulks.

Compared with the hybrid organic–inorganic perovskites, the all-inorganic perovskites CsPbX_3_ have enhanced stability and outstanding electronic properties. However, the CsPbX_3_ perovskite films fabricated by the spin-coating method contain defects of pinholes and voids, which affects the lasing performance seriously. To solve this problem, Li et al. introduced ZnO nanoparticles into the CsPbBr_3_ films [98]. They first prepared a CsPbBr_3_:ZnO precursor solution containing CsBr, PbBr_2,_ and ZnO nanoparticles in the N_2_ glovebox. They then spin-coated the precursor on the glass substrates by the one-step method. The chlorobenzene solvent was used as an anti-solvent to wash out the solvent DMSO to induce the perovskite fast crystallization. After annealing for 30 min, the CsPbBr_3_:ZnO films were fabricated. Using an fs laser system as the pumping source, the enhanced single-photon-pumped and two-photon-pumped ASE with thresholds of ~0.207 mJ/cm^2^ and ~0.569 mJ/cm^2^ were observed. The enhanced ASE came from the smaller crystal grains, the decoration of the ZnO, and a more compact surface. The perovskite films synthesized by the spin-coating method consisted of disordered nanoparticles and microparticles. Since no cavity boundaries were designed around these films, the laser emission was considered RL due to the disordered nature of the films. Duan et al. studied the miscellaneous lasing actions in MAPbBr_3_ perovskite films and proved that in addition to the random lasing, whispering-gallery-mode lasing could also generate in perovskite microparticles with their internal resonances instead of multiple scattering among them [99]. The miscellaneous lasing behaviors can help to understand the lasing actions in complex lead halide perovskite systems. 

Consistent with the 2D perovskite materials are the inherent multiple quantum well (QW) structures. They have many advantages compared to 3D materials, such as quantum tunable optoelectronic properties and photo- and chemical stabilities. In 2020, Liang’s group fabricated a long-chain organic diammonium spacer-assisted 2D hybrid-perovskite FA-(N-MPDA)PbBr_4_ single-crystal microrods by the slow evaporation at constant temperature method [100]. The single-crystal microrods exhibited high crystallinity with a well-defined rod shape. Through pumping by a ps laser source, the high-quality RL with a threshold of 0.5 µJcm^−2^ and narrow lasing linewidth of 0.1 nm was observed. The Q factor reported here is 5350, which is the highest value in perovskite RLs. The low threshold and high Q factor are attributed to the long mean free path, strong quantum confinement, and the large exciton binding energy of 2D single-crystal perovskite microrods. The 3D MAPbBr_3_ single-crystal random lasing at a low temperature of 4K was also studied [101]. In the MAPbBr_3_ single-crystal, the random resonator is formed by the crystal cracks and inhomogeneities. 

RLs do not rely on well-designed cavities and can be simply generated by the multiple scattering in the gain medium. The mode numbers, wavelengths, Q factors, and directionalities are random and hard to control, which hinders their practical applications. In 2019, Song’s group demonstrated a highly directional perovskite-based surface-emitting RL with lasing divergence angle <3–5° by combing the strong scattering perovskite film and planar microcavity [85]. In this work, the directionality of low-spatial-coherence RL was improved by DBR mirrors. The MAPbBr_3_ perovskite precursor solution was first spin-coated onto one of the mirrors. After annealing at a temperature of 80 °C for 10 min, the MAPbBr_3_ film was formed. Subsequently, another DBR mirror covered the perovskite film face to face, forming DBR−perovskite−DBR RL. The obtained surface-emitting RL thresholds and FWHM were approximately 25 μJ/cm^2^ and 1 nm, respectively. Using these directional RLs as light sources, the spectral-free imaging systems were shown. Bouteyre et al. designed an Ag/PMMA/MAPbBr_3_/Bragg mirror structure and realized a directional RL [90]. The thickness of the MAPbBr_3_ and PMMA films was 100 nm and 350 nm, respectively. The coupling of the lasing emission with the Bragg mirror guaranteed the tunable laser direction. 

The toxicity of lead-based perovskites is a problem for their application. Some methods have been proposed to fabricate lead-free optoelectronic devices. In 2021, Wu et al. fabricated CsSnI_3_ films and demonstrated a CsSnI_3_ perovskite RL operated at room temperature in ambient air [91]. In this work, the CsSnI_3_ film was synthesized by the chemical vapor deposition method. The CsI and SnI_2_ powders were used as precursors. The laser threshold was approximately 18 mJ/cm^2^. The distinct cavity length at different detected directions was analyzed by employing the FFT method. Suárez et al. further fabricated high-quality FASnI_3_ lead-free perovskite thin films and demonstrated a low-threshold flexible RL [92]. The laser structure consists of a PMMA/FASnI_3_ bilayer structure deposited on a flexible PET substrate. The high quality of FASnI_3_ films and optimized design waveguide structure made the lasing threshold as low as 300 nJ/cm^2^. 

## 4. Perovskite RL Applications

With outstanding performances, perovskite RLs have great potential in a wide range of applications, such as laser illumination, speckle-free imaging, display, and sensing. To date, some attempts have been conducted.

**Figure 5:**
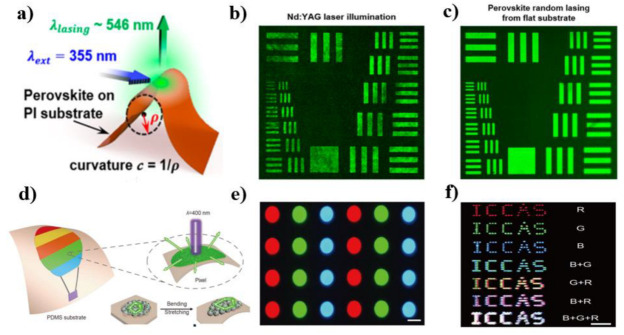
(**a**) Schematic illustration of excitation and emission of a flexible perovskite film laser under bending conditions. Optical imaging of Air Force resolution test chart via (**b**) Nd:YAG laser and (**c**) perovskite RL from the flat substrate. The images (**a**–**c**) adapted with permission from Ref. [84]; Copyright 2019 American Chemical Society. (**d**) Design principle of the flexible laser display based on RLs. (**e**) Fluorescence microscopy images of the periodic RGB emissive arrays under UV light radiation. (**f**) Far-field photograph of the “ICCAS” patterns of pixel arrays comprising different microlasers. The images (**d**–**e**) were adapted with permission from Ref. [74]; Copyright 2021 Science China Press and Springer-Verlag GmbH Germany, part of Springer Nature.

RLs are one class of innovative speckle-free intense light sources for advanced imaging apparatus because they combine the two advantages of high light output and low spatial coherence. In 2019, the perovskite RL for speckle-free imaging application was first reported by Wang et al. [84]. They fabricated a flexible perovskite RL by embedding curved MAPbBr_3_ perovskite thin film on the flexible polyimide substrate. They quantitatively analyzed the degree of spatial coherence and imaging capability of the MAPbBr_3_ perovskite RL. The results showed that the first-order spatial coherence of RL was less than 0.12, which was approximately one order lower than conventional Nd-YAG lasers. The image test further proved that perovskite RL was the ideal light source for high-quality speckle-free imaging (Figure 5a–c). Zhang et al. further demonstrated a miniaturized perovskite optical fiber RL for speckle-free imaging [86]. In this work, the MAPbBr_3_ perovskite films on the optical fiber’s end-face were first fabricated using the dip-coating method. The fabrication process included two steps. The optical fiber tip was first dipped into the PbBr_2_/DMF solution to obtain a layer of PbBr_2_ on the fiber facet. Afterward, the optical fiber tip was dipped into the MABr/isopropanol solution. After annealing at 70 °C for 20 min, the MAPbBr_3_ films on the optical fiber’s end-face were obtained. Using a 150-fs laser as a pump source, random lasing with a threshold of 32.3 μJ/cm^2^ was clearly observed. The divergence angle of the obtained laser beam was less than 60°. This directional RL is caused by the lower absorption in thinner films with small divergence angles and the refraction at the interface between the hemispherical perovskite film and air. The spatial coherence of the lasing was measured as γ = 0.6279, which was lower than that of traditional 532-nm continuous-wave (CW) lasers (γ = 0.9344). Moreover, the speckle contrast of RL was approximately 15 times lower than traditional lasers. In 2020, the perovskite RL was used for the holographic image projection by Mallick et al. [87]. They fabricated a perovskite–polystyrene 10 wt%-based RL using a solvent-engineered process. The polystyrene on perovskite films induced a low laser divergence angle of ≈10^0^. Using this RL as a holographic image light source, the authors produced a significant speckle-free holographic image projection. 

Perovskite materials are sensitive to environments such as water, oxygen, and temperature. Using these properties, Li et al. fabricated perovskite CsPbBr_3_ nanorods with an optical gain coefficient as high as 954 cm^−1^ and proposed a sensitive humidity sensing based on perovskite RL for the first time [72]. In contrast to LED and other incoherent luminescence, lasing is much more sensitive to the surrounding environmental conditions. The perovskite RL sensor could monitor the humidity from 30–72% RH. In 2021, Zhao’s group demonstrated full-color perovskite RL arrays for flexible laser display [74]. In this work, the CsPbX_3_ QD/SiO_2_ composites were used as gain media and multiple scattering structures to construct red, green, and blue-emissive micro RLs. The microlasers were precisely positioned into micro templates, forming RGB display pixels (Figure 5d–f). The flexibility and omnidirectional emission properties of RL enable the display device to maintain stable performance under deformation. 

The perovskite RLs mentioned above are based on the optical-pumping source, which hinders their practical application. In most RL applications, electrical pumping is essential. Nevertheless, it has not been realized so far. The optical gain for perovskites requires the excitation of more than one. To achieve population inversion under electrical pumping, high electric current injection is needed. However, the high current will damage the laser device by Joule heating. Moreover, the multiple excitons undergo rapid non-radiative Auger recombination, prohibiting the sustained population inversion.

## 5. Conclusions and Outlook

In this work, we overview the recent developments of perovskite RLs. We start with the summary of the hybrid organic–inorganic perovskite QD and the all-inorganic perovskite QD-based RLs. We then discussed the perovskite film RLs. The perovskite nano-/microstructure fabrication process, the novel RL structure, and lasing properties are also discussed in detail. In the last part, we review the frontier applications of perovskite RLs in laser illumination, speckle-free imaging, laser display, and sensing. 

Halide perovskite was proven to be in a class of high-performance gain materials due to its high performance in broadband absorption, single/multiphoton absorption coefficient, low non-radiative recombination, and tunable emission wavelength. In addition, High-quality perovskite QDs can be in situ fabricated in many matrixes, such as polymer film, glass, and MOFs. The perovskite films can also be fabricated by a simple one-step or two-step deposition process. The convenient solution-processability makes perovskite RL integrate on different substrates easily. Perovskite RLs have become a hot research field in the past few years. Significant advantages have been achieved. However, the solution-processed perovskite RLs are still in the initial stage toward practical applications. Some of the following challenges remain. 

First, the biggest challenge for perovskite RL is the high threshold. The expensive fs lasers are used as pump sources in most previous works. To date, the CW laser pump or the electrically pumped perovskite RL has not been reported. Perovskites are potential gain media to achieve CW RL laser or electricity-driven RL. The perovskite Fabry–Pérot (FP) and distributed feedback (DFB) CW lasers using CsPbBr_3_ nanowires, MAPbI_3_ films, and MAPbX_3_/polymer composite films as gain media have been realized [48,49,102,103,104,105,106,107,108,109,110]. With perovskite RLs, there are three factors influencing the CW laser threshold: the optical gain, the heat effect, and random cavity structure quality. The optical gain defines the ability of the gain media to achieve optical amplification. For solution process semiconductors, such as perovskites and II-VI QDs, the optical gain normally requires an average number of excitons greater than one. Perovskite materials show high gain absorption coefficients. The net gains of CsPbBr_3_ QDs and MAPbBr_3_ film were measured as 450 cm^−1^ and 3200 cm^−1^, respectively [111,112], which guaranteed the possibility of achieving CW RLs. It should be noted that the gain properties are dependent on the material temperature. High temperatures induce large non-radiative Auger recombination, which increases the lasing threshold. High-power laser injection, quantum defect, and non-radiative recombination generate substantial heat. Moreover, the strong multiple scattering in perovskite RLs produces more heat. The optical cavity quality is another important factor affecting the laser threshold. The optical feedback of RLs comes from the multiple scattering of the random media. However, the physical process in disordered systems is complex. To date, there has been a lack of specialized studies on the relationship between perovskite RL thresholds and cavity properties. 

Second, the poor stability of perovskite RLs is another challenge. Perovskite materials are easy to degrade under conditions of irradiation, moisture, and oxygen. How to solve poor stability is a long-lived hot topic before their practical applications. One way is to develop new organic or inorganic ions for perovskites and control their morphology into low-dimensional structures. The coating is another effective way. Indeed, many matrixes, such as porous networks, TeO_2_/SiO_2_ glass, polymer films, and MOFs, have been pursued [22,64,66,67,68,75,79]. 

Third, the toxicity of Pb-based perovskites limits their real laser application. The solution is to develop Pb-free perovskites by substituting Pb with low-toxicity Sn, Ge, or other elements. The Pb-free CsSnI_3_, FASnI_3_ perovskite, and their RLs were reported. However, the optical property and stability of Sn/Ge-based perovskites are not as good as Pb-based perovskites. Much more work should be conducted to improve their performance. 

Last, it remains a challenge to control the perovskite RL laser emission. Because of the lack of well-designed cavities, the lasing intensity, wavelength, mode number, and directionality are random and hard to control. It is harmful to their practical application, especially in the laser display field. Combing the scattering cavity with a conventional FP or DFB cavity is a solution. By taking advantage of the selection mode property of conventional cavities, it is possible to achieve high directional low spatial coherence RLs. 

## Figures and Tables

**Table 1:** Selected representative results of RLs from halide perovskite gain media.

Authors	Description	Pump Type	Pump Wavelength	Emission Wavelength	Thresholds	FWHM	Operation Condition	Publication Date (Year)
Perovskite QD RLs								
Yakunin et al. [61]	CsPbBr_3_ QDs	fs	400 nm	~483 nm	~5 μJ/cm^2^	0.14 nm	RT	2015
Liu et al. [62]	MAPbBr_3_ QDs	fs	400 nm/800 nm	~548 nm	60 μJ/cm^2^/4.4 mJ/cm^2^	1 nm	RT	2016
Hu et al. [63]	MAPbBr_3_ QDs	ps	374 nm	540 nm	~34.5 μJ/cm^2^	0.4 nm	RT	2017
Yuan et al. [64]	CsPbBr_3_ QD-embedded glass	fs	800 nm	530–540 nm	200 μJ/cm^2^	<1 nm	77 K	2018
Roy et al. [65]	MAPbBr_3_ QDs/GNW composite	ps	374 nm	530 nm	1 nJ/cm^2^	0.4 nm	RT	2018
Liu et al. [66]	CsPbBr_3_-SiO_2_ sphere	fs	800 nm	537 nm	665 μJ/cm^2^	<1 nm	RT	2019
Tang et al. [67]	CsPbBr_3_ QDs	fs	800 nm	540 nm	9.54 mJ/cm^2^	0.49 nm	RT	2019
Yang et al. [68]	FAPbBr_3_/A-SiO_2_ composites	fs	800 nm	~550 nm	413.9 μJ/cm^2^	~1307	RT	2020
Zhu et al. [69]	CsPbBr_3_ QDs	electron beam	-	~540 nm	3kV	0.9 nm	RT	2020
Liu et al. [70]	Cs_4_PbBr_6_ monolith ceramics	fs	400 nm/800 nm	~541 nm	25.98 μJ/cm^2^ 719 μJ/cm^2^	<1 nm	RT	2020
Yang et al. [71]	CsPbBr_3_ QDs	fs	800 nm	535 nm	254 μJ/cm^2^	0.3 nm	77 K	2020
Li et al. [72]	CsPbBr_3_ nanorods	fs	355 nm	532 nm	18.8 μJ/cm^2^	0.2 nm	RT	2020
Li et al. [73]	CsPbBr_3_ QD/SiO_2_ nanodots	fs	800 nm	533 nm	0.91 mJ/cm^2^	0.3 nm	RT	2020
Hou et al. [74]	CsPbX_3_ QD/SiO_2_ composites	fs	400 nm	RGB	52.1–72.3 μJ/cm^2^	-	RT	2021
Xu et al. [75]	MAPbX_3_ QDs@Pb-MOFs	ps	490 nm	Multicolar 520–780 nm	0.38 mJ/cm^2^	1.2 nm	RT	2021
Wang et al. [22]	CsPbX_3_@glass@ SEBS	fs	800 nm	~523 nm	0.16 mJ/cm^2^	-	RT	2021
Xing et al. [76]	Ag NW coupled CsPbBr_3_ QDs	fs	400 nm	~520 nm	~34.5 μJ/cm^2^	~2.5 nm	RT	2021
Gao et al. [77]	CsPbBr_3_ QDs	fs	800 nm	537 nm	190 μJ/cm^2^	1 nm	RT	2021
Jin et al. [78]	CsPb(Br/I)_3_ QD glasses	fs	800 nm	590 nm	0.79 mJ/cm^2^	3 nm	93 K	2021
Xiong et al. [79]	CsPbBr_3_ QD/SiO_2_ composites	fs	800 nm	527 nm	79.81 μJ/cm^2^	0.4 nm	RT	2022
Perovskite film RLs								
Dhanker et al. [60]	MAPbI_3_ planar microcrystal networks	ns	355 nm	~785 nm	200 μJ/cm^2^	<0.5 nm	RT	2014
Kao et al. [80]	MAPbI_3_ film	ns	355 nm	~745 nm	~230 μJ/cm^2^	-	77 K	2016
Xu et al. [81]	FAPbBr_3_/PEOcomposite film	ns	1064 nm	538 nm	1.1 mJ/cm^2^	0.4 nm	RT	2018
Weng et al. [82]	MAPbBr_3_ film	fs	1300 nm	~547 nm	27 mJ/cm^2^	5 nm	RT	2018
Safdar et al. [83]	MAPbI_3_ films	ns	532 nm	780 nm	10 μJ/cm^2^	5 nm	RT	2018
Wang et al. [84]	MAPbBr_3_ film	ns	355 nm	~546 nm	2.5 mJ/cm^2^	1.8 nm	RT	2019
Liu et al. [85]	MAPbBr_3_ film	ps	400 nm	540–560 nm	~25 μJ/cm^2^	<1 nm	RT	2019
Zhang et al. [86]	MAPbBr_3_ film on fiber facet	fs	400 nm	552 nm	32.3 μJ/cm^2^	4 nm	RT	2020
Mallick et al. [87]	MAPbBr_3_ film	ns	355 nm	~550 nm	~6.6 mJ/cm^2^	<1 nm	RT	2020
Hong et al. [88]	MAPbBr_3_ films	ns	355 nm	~550 nm	0.9 mJ cm^2^	<1 nm	RT	2020
Mallick et al. [89]	MAPbBr_3_ films	ns	355 nm	546 nm	2.6 mJ/cm^2^	~3 nm	RT	2020
Bouteyre et al. [90]	MAPbBr_3_ film	fs	405 nm	545 nm	104 μW	0.7 nm	RT	2020
Wu et al. [91]	γ-CsSnI_3_ films	ps	355 nm	950–960 nm	18 mJ/cm^2^	0.3 nm	RT	2021
Suárez et al. [92]	FASnI_3_ films	ns	532 nm	890 nm	300 nJ/cm^2^	0.8 nm	RT	2022
Hu et al. [93]	MAPbBr_3_ films	fs	400 nm	~550 nm	12.3 μJ/cm^2^	7.7 nm	RT	2022

Note. RT represents room temperature.
